# Zirconia vs. Titanium Dental Implants: Primary Stability In-Vitro Analysis

**DOI:** 10.3390/ma14247886

**Published:** 2021-12-20

**Authors:** Nerea Arlucea, Aritza Brizuela-Velasco, Markel Dieguez-Pereira, Miquel Punset, Meritxell Molmeneu, Fernando Sánchez Lasheras, Hector deLlanos-Lanchares, Ángel Álvarez-Arenal

**Affiliations:** 1 Department of Prosthodontics and Occlusion, School of Dentistry, University of Oviedo, c/Catedrático Serrano s/n, 33006 Oviedo, Spain; nerearlucea@gmail.com (N.A.); aritzabrizuela@hotmail.com (A.B.-V.); markeldieguez@hotmail.com (M.D.-P.); llanoshector@uniovi.es (H.d.-L.); arenal@uniovi.es (Á.Á.-A.); 2 Biomaterials, Biomechanics and Tissue Engineering Group, Department of Materials Science and Metallurgy, EEBE, Technical University of Catalonia (UPC), 08034 Barcelona, Spain; miquel.punset@upc.edu (M.P.); meritxell.molmeneu@upc.edu (M.M.); 3 UPC Innovation and Technology Center (CIT-UPC), Technical University of Catalonia (UPC), C. Jordi Girona 3-1, 08034 Barcelona, Spain; 4 Barcelona Research Centre in Multiscale Science and Engineering, Technical University of Catalonia (UPC), Av. Eduard Maristany, 10-14, 08019 Barcelona, Spain; 5 Department of Mathematics c/Federico García Lorca 18, University of Oviedo, 33007 Oviedo, Spain; 6 Instituto Universitario de Ciencias y Tecnologías Espaciales de Asturias (ICTEA), c/Independencia 13, 33004 Oviedo, Spain

**Keywords:** insertion torque, micromotion, resonance frequency analysis, titanium dental implants, zirconia dental implants

## Abstract

The present experimental trial uses two types of dental implants, one made of titanium (Ti_6_A_l4_V) and the other one of zirconia (ZrO_2_), but both of identical design, to compare their stability and micro-movements values under load. One of each type of implant (*n* = 42) was placed into 21 cow ribs, recording the insertion torque and the resonance frequency using a specific transducer. Subsequently, a prosthetic crown made of PMMA was screwed onto each of the implants in the sample. They were then subjected to a static compression load on the vestibular cusp of the crown. The resulting micromovements were measured. The zirconia implants obtained a higher mean of both IT and RFA when compared with those of titanium, with statistically significant differences in both cases (*p* = 0.0483 and *p* = 0.0296). However, the micromovement values when load was applied were very similar for both types, with the differences between them (*p* = 0.3867) not found to be statistically significant. The results show that zirconia implants have higher implant stability values than titanium implants. However, the fact that there are no differences in micromobility values implies that caution should be exercised when applying clinical protocols for zirconia based on RFA, which only has evidence for titanium.

## 1. Introduction

The most commonly used alloy in the manufacture of dental implants is Ti_6_Al_4_V (Ti), which is also the most strongly supported by scientific evidence [1]. However, at present, other titanium alloys and materials are used at the clinical level, particularly zirconia. This material is used in the form of zirconium oxide (ZrO_2_), usually yttrium-stabilized, and is obtained from a reductive oxidation process. Its advantages, according to some authors [2], are biocompatibility and mechanical properties superior to those of Ti.

Certain clinical trials have shown the clinical and histological advantages that are achieved with ZrO2 implants when compared to Ti implants. For example, Bienz et al. [3] observed that lower plaque and bleeding scores were detected for zirconia implants under experimental mucositis conditions, in agreement with other clinical studies with similar objectives [4]. However, another clinical study did not find differences in relation to the health of the peri-implant soft tissues [5] and in fact the results of a cross-sectional study did not show important differences in relation to the expression of pro-inflammatory cytokines and bone metabolism mediators between zirconium and titanium abutments [6].

On the other hand, beyond the inflammatory response, it is foreseeable that two materials of such different stiffness as titanium and zirconia have different biomechanical behavior in relation to the supporting bone. In this sense, some studies using finite elements analysis have found differences in the load transfer in relation to the Young’s modulus of the material from which the implant is manufactured [7]. Likewise, an animal model trial observed different percentages of bone to implant contact after the osseointegration period in implants made of alloys with different elasticity [8].

Regardless of the material used, the osseointegration of a dental implant is a process that can be compared to the primary healing of a fracture, and in both a series of biological and mechanical requirements must be met. Among the latter, the control of micromovement between the interface of the implant surface and the bone apposition is particularly important. Micromovement of more than 150 µm at this level would be likely to rupture the newly formed capillaries, which would lead to the slowing or failure of the bone regeneration process, setting in motion a reparative process based on the apposition of fibrous tissue [9].

In implantology, the term “primary stability” is used to describe the implant’s lack of mobility when a load is applied [10]. Ultimately, both of the aforementioned concepts are inversely related: greater primary stability of the implant means less micromovement between the interface of the implant surface and the bone apposition. For this reason, it is essential that clinical procedures measure primary stability [11].

Insertion torque (IT), expressed in Ncm, is one of the most commonly used methods for this purpose. IT can be considered as the resistance encountered during the insertion of the implant into the surgically shaped bone bed, when advancing apically and rotating on the longitudinal axis. There are two problems with this method. First, there is the paradox that excessive IT can exceed the bone’s resistance to fracture, in which case stability could be compromised or, at the very least, not increased [12]. Secondly, it exemplifies a certain type of inertia, and in chewing where Bennett movement is limited, such a rotational force is not applied and resistance to it may not be key [13]. On the contrary, the resultant forces can be more accurately described as lateral displacement, for which other primary stability measurement methods are much more specific, notably resonance frequency analysis (RFA). RFA provides objective measurements of implant stability in a non-invasive way on the bone-implant interface [11]. For this purpose, a specific transducer is screwed to the connection of the implant, which is electromechanically stimulated, detecting the natural vibration frequency of the implant within the bone. The results of the resonance frequency analysis are transformed into implant stability quotient (ISQ) values. Certain studies show, on the one hand, that RFA is capable of describing the stiffness of the bone support [10], it correlates with the lateral displacement of the implant upon load application [14,15], and more importantly, it presents an inversely linear relationship with micromovement [13].

Several clinical studies have used the two aforementioned stability measurement methods (IT and RFA) to try to establish a predictive value for the possibility of osseointegration, occasionally in highly complex protocols such as immediate loading [16,17]. Even when other variables may be involved (prosthetic splinting, occlusal adjustment, etc.), there is some consensus that values equal to or greater than 30 Ncm and 70 ISQ could be adequate when making the decision to immediately load an implant [18].

Ultimately, the clinician may select a protocol and can influence the stability values by considering or managing the variables on which stability depend: bone quality, implant design, and surgical technique. Yet, there is no literature regarding the effect of the implant material. The Young’s modulus of a Zr implant is practically double that of a Ti implant (200 vs. 110 GPa respectively [19]). Such a marked difference in elasticity can influence its mechanization in the bone and, ultimately, the expected IT and RFA stability values.

The objective of this experimental in vitro study is to determine the impact of the elastic properties of the implant material (Zr or Ti) on dependent variables in relation to primary implant stability such as IT, RFA, and micromovement. The null hypothesis proposed is that the properties of the material used do not influence the stability of the implant.

## 2. Materials and Methods

### 2.1. Sample

**Figure 1:**
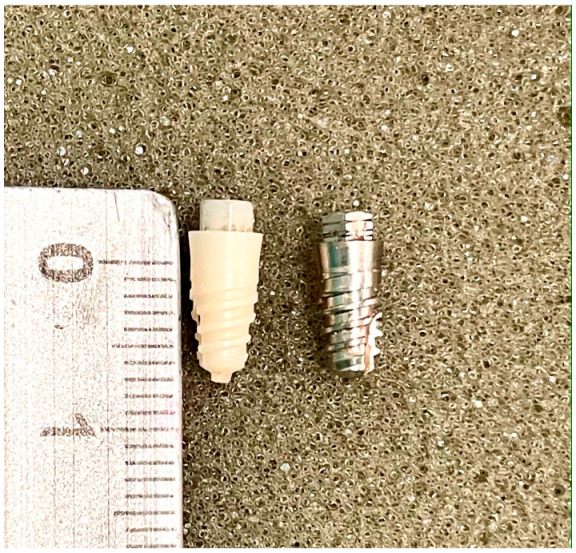
Klockner External Hex SK2 implants, Zr and Ti, both with 3.8*8 buffed surface (Soadco S.L., Escaldes Engordany, Andorra).

The sample consisted of the placement of 21 Zr implants (test) and 21 Ti implants (control group) (*n* = 42), in another 21 fragments of fresh cow ribs destined for human consumption. We based our selection of the sample size on studies of similar design and objectives [13,14,15]. The most central part, close to the sternum, was used in all cases in order to have type III bone quality under the Lekholm and Zarb classification [20]. All pieces were cut to a length of 15 cm, and the periosteum was removed. The ribs thus prepared were subjected to a preparation and conservation treatment based on immersion in 50% ethanol and 50% saline at room temperature, to be rehydrated with saline prior to use, according to the Tricio protocol [21]. Klockner SK2 implants (Soadco, Escaldes-Engordany, Andorra) were used, connected by an external hexagon 3 mm wide and 1.8 mm high, smooth collar 1.5 mm high and 4.2 mm platform diameter, bone body of 3.8 mm diameter and 8 mm in length, and machined surface, without additional modifications (Figure 1).

### 2.2. Working Model and IMPLANT Stability Measurement (IT and RFA)

The ribs, once rehydrated, were embedded in a Snow White Type 2 Plaster base (Kerr, Orange County, CA, USA) to facilitate their handling during the surgical phase, and to avoid unwanted micromovement during the application of load creep. Three polymethyl methacrylate (PMMA) crowns were made, with a morphology typical of lower premolars, cemented with a dual-cure material (Clearfil DC Core Plus, Kuraray Noritake Dental Inc., Osaka, Japan) on an anti-rotational grade 5 titanium abutment to be screwed to the implants of the sample.

One of each type of implant was placed in the 21 ribs. The implant beds were prepared using the surgical kit and following the protocol established by the manufacturer: Ø 1.8 initial drill, Ø 2.3 pilot drill, Ø 2.8 profile drill, and Ø 3.3 final drill. The separation between both beds (10 mm between centers) was standardized, and the position of each implant was randomized using a biased coin.

**Figure 2:**
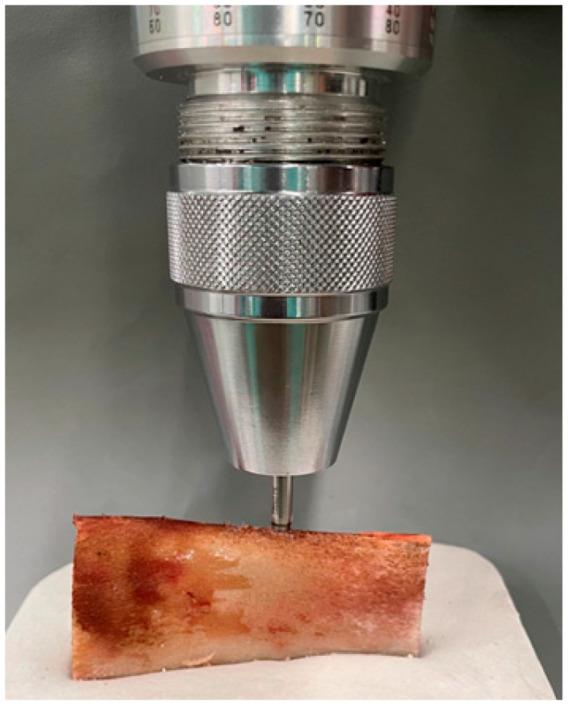
Placement of the implant, in the surgically shaped bone bed, using a calibrated torque meter, to record the insertion torque in Ncm.

A previously calibrated BTG90CN analog torque meter (Tohnichi, Tokyo, Japan) was used (Figure 2), which measured the insertion torque (in Ncm) required to bring the implant to its final position, which in all cases was left with the smooth collar of the implant placed supra-crestally.

**Figure 3:**
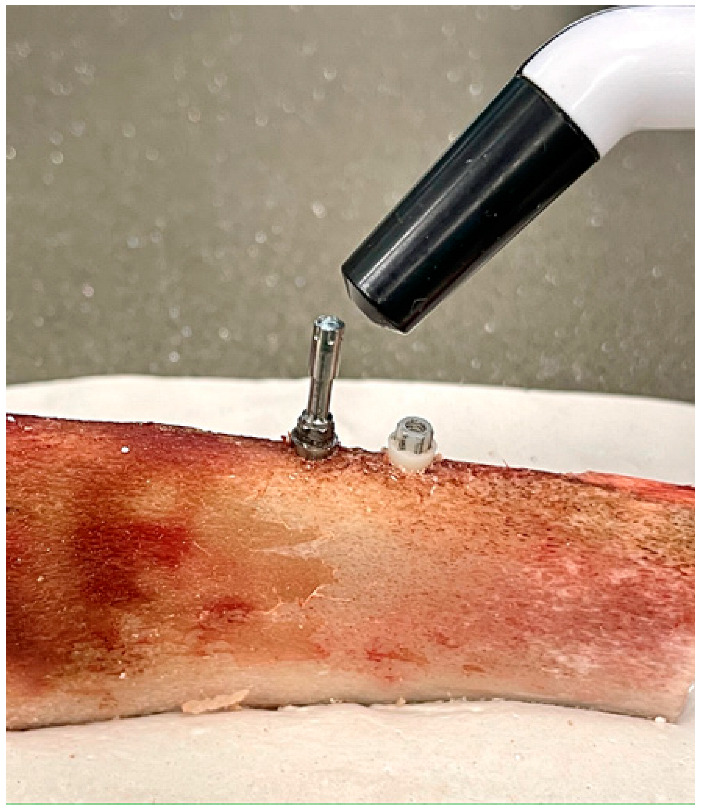
Measurement of implant stability by resonance frequency analysis using a Penguin and its Multipeg transducer screwed to the TI implant, expressed in ISQ values. On the right, the Zr implant placed in the same rib can be seen.

Subsequently, the ISQ values were measured with a Penguin RFA (Integration Diagnostics Sweden AB, Gothenburg, Sweden) using a Multipeg transducer screwed to the implants with a manual preload, following the manufacturer’s recommendations (Figure 3). Finally, the PMMA crown was screwed onto each implant in the sample, using a torque wrench, applying a preload of 15 Ncm in both groups, also as recommended by the manufacturer.

### 2.3. Loading Test and Recording Micromovement

**Figure 4:**
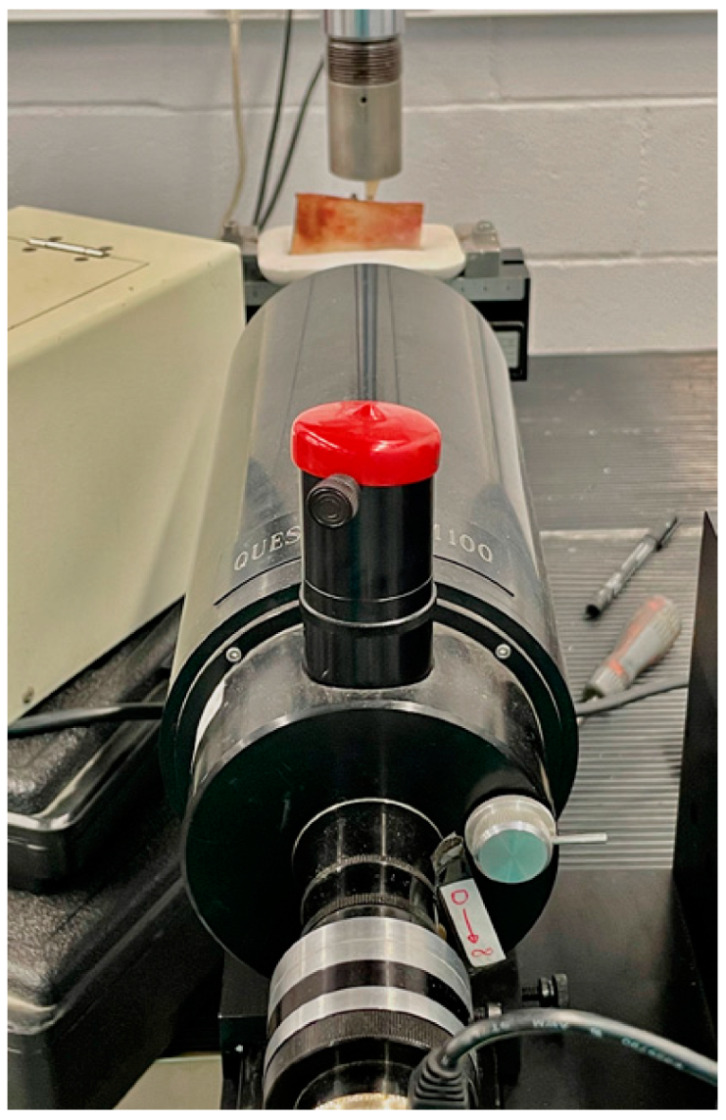
Questar QM-100 long-distance microscope focused on one of the implants in the sample. In the background you can see a rib mounted on its plaster base and mechanized on the base of the load creep machine.

A Bionix 358 servohydraulic mechanical testing machine (MTS Sensor Technologie GmbH & Co. KG, Lüdenscheid, Germany) with a 2.5 kN load cell was used for the micromovement study. The work models (ribs with one of each type of implant, with their screw-retained PMMA prosthetic crown) were positioned on the grip at the base of this equipment. To conduct the study, an upper mechanical clamp is prepared to apply tension at a 6-degree incidence angle to the occlusal plane of the premolar (Figure 4). All the crowns were positioned with the same orientation and with the same load application direction.

**Figure 5:**
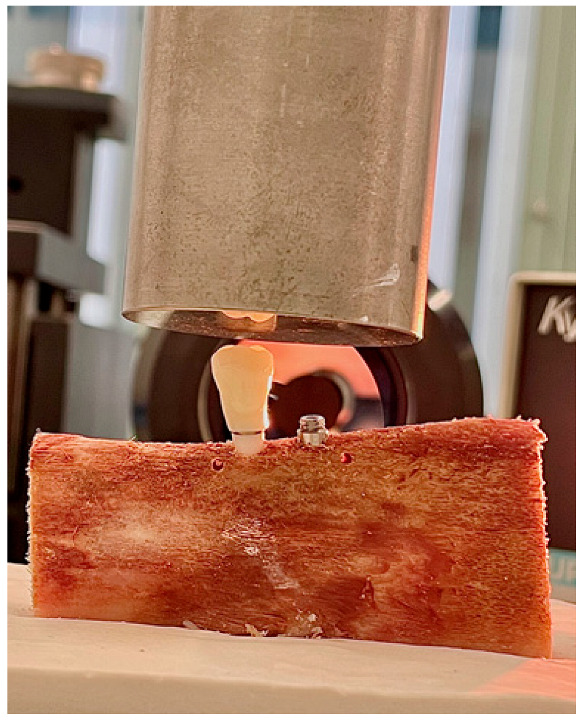
Detail of the PMMA crown screwed on the external hexagon of the Zr implant, and mounting on the MTS Bionix 358 test machine. In the background you can see the lens of the Questar QM-100 long-distance microscope.

Subsequently, a compression force was tested on each implant/crown of the sample, applying a ramp from 0 to 50 N at a load application rate of 1 N/s, and a constant final application of 50 N for 10 s. In order to evaluate the micromovement, the displacement of the smooth collar of the implant was evaluated based on a reference mark added to its surface. Images were acquired at baseline with F = 0_1_ (initial), F = 1 (50 N), and at F = 0_2_ (0 N, unloading), using a Questar QM-100 microscope (long distance microscope) (Figure 5) with a resolution of 2 µm (Seven Astro-Optics Division, Laurel, MD, USA), and analyzed using AnalySIS getIT software (Olympus, Tokyo, Japan).

**Figure 6:**
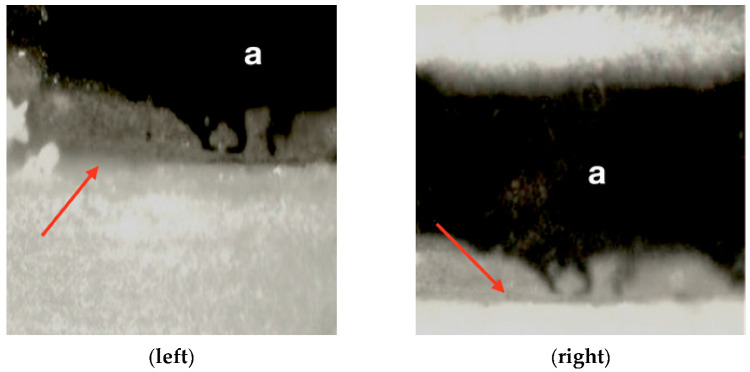
Detail of the microscopic analysis of the micromovement test (**left**) moment prior to loading (F = 01), (**right**) moment during the application of the 50 N load (F = 1). In both images, the “a” indicates the reference mark added to the abutment and the red arrows indicate the transition area between the prosthetic abutment and the smooth collar of the implant.

Having obtained the images, the distance was calibrated using a standard for optical microscopy and the displacements in the orthogonal X axis were measured using ImageJ software (National Institute of Health, Bethesda, USA) under the three load levels described (F = 0_1_, F = 1, and F = 0_2_) (Figure 6).

### 2.4. Statistical Analysis

The Anderson–Darling test was applied to the results of the dependent variables to determine the normality of the distribution of the samples.

The Mann–Whitney test was used to calculate the possible relationship between the different variables studied (IT, RFA, and micromovement). If *p* < 0.05, differences in the comparison of the dependent variables are considered statistically significant.

Finally, the Pearson correlation coefficient was applied to corroborate the relationship between ISQ and micromovement, according to the evidence available in the literature [13,14,15]. For a result equal to 0, these variables are considered unrelated, and they will be considered related if it is higher or lower than 0, directly or inversely, based on whether the coefficient acquires a positive or negative value, respectively.

## 3. Results

Table 1 shows the descriptive statistics of the study’s dependent variables (mean and SD), the result of the statistical significance of the difference between variables when applying the Mann–Whitney test, and the Pearson correlation coefficient between the ISQ and micromovement variables.

Regarding IT, we see a value above the mean achieved in the Zr implant group (29.90 Ncm) when compared with Ti implants (23 Ncm). This difference turned out to be statistically significant with *p* < 0.05 (*p* = 0.0483). Regarding RFA, again, the ISQ mean values were higher for the Zr implants than the Ti implants (69.429 versus 62.095 respectively), and these differences were also statistically significant with *p* < 0.05 (*p* = 0.0296).

However, comparing the mean micromovement values of the implants when load is applied at F = 1 (50 N) in the orthogonal X axis, the values barely reach a difference of one micron (93.5 for Ti and 94.450 for Zr) and are not statistically significant (*p* = 0.3867) according to the Mann–Whitney test.

**Figure 7:**
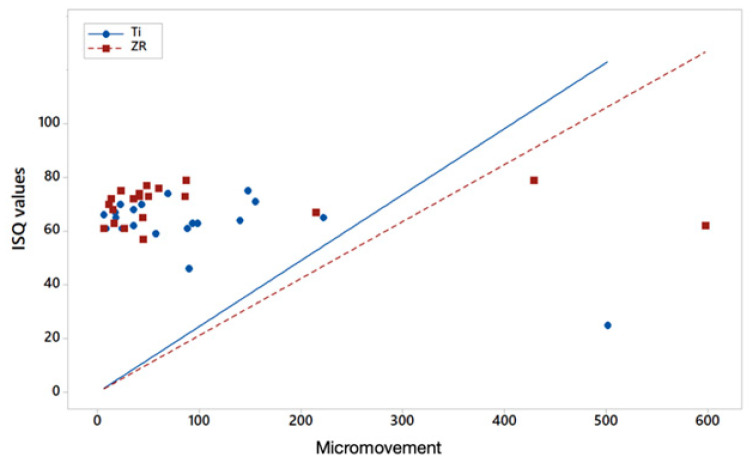
Scatter plot of ISQ values versus micromovements (μm) for titanium and zirconium implants.

Regarding the correlation between ISQ and micromovement, for both types of implant tested (Zr and Ti) there is an inverse linear relationship between both variables, so it is to be expected that the higher the ISQ, the lower the micromovement. However, the degree of correlation is higher for Ti (−0.673) and weaker for Zr (−0.007) (Figure 7).

## 4. Discussion

The objective of this experimental in vitro study was to establish the differences in primary stability between two different implant materials (Zr and Ti) in relation to dependent variables, such as IT, RFA, and micromovement.

For this purpose, a cow rib bone was used as a study model, attempting to simulate the conditions of a maxillary alveolar process. The high SDs found in some of the dependent variables, especially micromovement, demonstrate the lack of homogeneity of the density and presence of cortical bone support. Regardless, this model has been used regularly in studies of similar design and different objectives [13,14,15] and it is closer to a living bone model than polyurethane resins [22,23].

However, the study is not without limitations. We based our choice of the applied static force (50 N–6°) on Watanabe’s study [24]. It is clear that this type of load cannot exactly reproduce the complexity of the resultant forces created during chewing, which also essentially constitute a dynamic load. On the other hand, in order to simplify the process and make it feasible, we have exclusively considered the displacement of the implant on the orthogonal X axis. However, we believe that this is logical, given that various studies have shown that RFA is related to lateral implant displacement upon application of load [14,15].

The two implants being evaluated, which constituted the independent variables, were made of materials with very different Young’s modulus values (Ti and Zr, 110 and 200 GPa respectively). However, as indicated above in Section 2, their designs and dimensions were identical, and care was even taken to remove surface roughness modifications. The surface of both types was machined, and their roughness therefore depended only on the morphology of the milling instrument. It was important to take this precaution so as not to create a confounding variable, as various studies have provided results with rougher surfaces generating a higher friction coefficient during implant insertion and tending to generate higher IT [23,24,25].

In the scientific literature currently available, we have not found studies comparing stability values between Zr and Ti implants using IT and RFA, with the exception of the study by Ibrahim et al. [26]. This study used an experimental in vitro test model similar to ours, and where appropriate, used polyurethane resin blocks. As in our study, Zr obtained higher ISQ and IT values than Ti. However, in this study, there was no homogeneity in the characteristics and designs of the implants being compared, which could constitute a potential covariate.

On the other hand, a recent experimental in vitro study using homogeneous polyurethan foam material, which simulated alveolar bone, concluded that the increasing density of the resin and under-drilling protocols were related to higher IT for internal connection zirconia implants. In that same study, with conventional drilling protocols in low and medium density support resins, conditions similar to those of our trial, they obtain IT averages ranging from 18.01 to 28.98 Ncm, values close to those of our averages in the zirconium implant (29.05 Ncm) [27]. Likewise, in an experimental study in an animal model (fox hound dogs), the authors tested three groups of zirconium implants and one titanium as control, all of them of different design and macrogeometry. In their results, they show that their group C (sandblasted zirconia plus all microgrooved) presents IT values higher than that those of titanium. Again, to the contrary of our study, not having taken into account the influence of the implant geometry on the IT, it may have been a confounding variable if not a covariate [28].

Besides, studies can be found in the literature, especially in animal models, that compare the two types of implants that we have evaluated (Ti and Zr). The dependent variable that is often analyzed is the Bone to Implant Contact (BIC). In this sense, Dubruille et al. [29], in their histomorphometric analysis of the implant bone interface, conclude that Zr implants obtain a mean BIC percentage higher than that of titanium. These results are consistent with the conclusions of other studies with similar design and objectives [30,31,32,33,34]. However, other studies show similar results in this regard for both materials [35,36,37]. In general, all of these studies recommend the need for well-designed long-term clinical trials to show peri-implant bone outcomes and the clinical performance of Zr implants.

It is also possible to find clinical studies comparing the two types of implants. Again, possible differences in primary stability are not evaluated and survival during the follow-up period is taken into account. Payer et al. [38] investigated two-piece zirconia and titanium implants loaded with cemented crowns. After a 30-month period, survival rates of 93.3% and 100%, respectively, were recorded. On the contrary, Ostman [39] retained removable hybrid dentures in 73 one-piece zirconia implants and 56 titanium implants in 24 edentulous patients. After 16 months, survival rates of 82.1% were recorded for titanium and 71.2% for zirconia, with the latter showing a considerable decrease in the survival rate. Grassi et al. [40] are performing a five-year clinical follow-up after the placement of single-piece Zr implants. As relevant data for our study, in order to achieve adequate primary stability, these were inserted with an insertion torque of 40 Ncm. The mean of our IT was 25 Ncm, and as with high torques there is the risk of implant fracture.

.Finally, despite the higher IT and RFA values of Zr implants with regard to micromovement, no statistically significant differences were found with those of Ti. In a study published by our own research group in 2015, with a design similar to the current one, we found an inverse linear relationship between ISQ values and the micromovement of the implants, in this case of titanium [13]. In this regard, these results were very similar to those found in the current study, where we have again found a strong correlation between the ISQ and micromovement of Ti implants (Pearson coefficient of −0.673 and −0.86 in the 2015 study). However, the inverse linear relationship between ISQ and micromovement of Zr implants is weak, −0.007. This weak correlation may be due to the elastic properties of Zr itself. One must consider that the RFA is relative to the mass and elastic properties of the material being analyzed. The difference between the mass of a Zr implant and a Ti implant is negligible due to the volume of the implant itself, but its Young’s modulus is practically double. Ultimately, our results allow us to suspect that Zr implants will tend to provide higher ISQ values not completely related to the stiffness of the supporting bone, but to its own intrinsic stiffness

The clinical relevance of the results of our study is that they lead us to recommend caution regarding protocols for Zr implants that are based on primary stability studies of Ti. Using the IT and ISQ means for Zr implants (29.9 and 69.4), they would be very close to the 30 Ncm and 70 ISQ values recommended by clinical studies in order to safely perform an immediate loading protocol [16,17]. However, according to our study, for Zr, the associated micromovement could present unsuitable values for osseointegration.

More in vitro and clinical studies are required, with designs providing more evidence to evaluate the behavior regarding the stability of implants made of highly rigid materials.

## 5. Conclusions

In view of our results and taking into account the limitations inherent to these types of studies, it can be concluded that Zr implants have significantly higher IT and RFA values than Ti implants, and in terms of micromovement they do not have significant differences when a load of 50 N is applied. Based on these results, it is not possible to completely reject the null hypothesis considered.

The clinical recommendation is, regarding Zr implants, to not apply decision-making protocols that are based on the primary stability of Ti implants.

## Figures and Tables

**Table 1:** Descriptive statistics (mean and SD) of the dependent variables analyzed (insertion torque, resonance frequency analysis, and micromovement) and statistical significance of the differences between independent variables (titanium and zirconia) * Statistically significant (α = 0.05; *p* < 0.05).

Variable	Anderson-Darling Test for Normality	Ti			Zr	
		N	Mean (SD)	*p*-Value M-W	N	Mean (SD)
Torque	AD = 1.69 *p* < 0.005	21	23 (7.029)	* 0.0483	21	29.05 (11.458)
ISQ	AD = 1.431 *p* < 0.005	21	62.095 (10.611)	* 0.0296	21	69.429 (6.630)
Micromov X	AD = 5.339 *p* < 0.005	21	93.5 (112.426)	0.3867	21	94.450 (152.761)
Pearson correlation coefficient ISQ–micromov			−0.673			−0.007

## Data Availability

The data presented in this study are available on request from the corresponding author.

## References

[1] Nicholson J.W. (2020). Titanium Alloys for Dental Implants: A Review. Prosthesis.

[2] Passos S.P., Nychka J.A., Major P., Linke B., Flores-Mir C. (2014). In Vitro Fracture Toughness of Commercial Y-TZP Ceramics: A Systematic Review. J. Prosthodont..

[3] Bienz S.P., Hilbe M., Hüsler J., Thoma D.S., Hämmerle C.H.F., Jung R.E. (2021). Clinical and histological comparison of the soft tissue morphology between zirconia and titanium dental implants under healthy and experimental mucositis conditions—A randomized controlled clinical trial. J. Clin. Periodontol..

[4] Blaschke C., Volz U. (2006). Soft and hard tissue response to zirconium dioxide dental implants--a clinical study in man. Neuro Endocrinol. Lett..

[5] Van Brakel R., Meijer G.J., Verhoeven J.W., Jansen J., De Putter C., Cune M.S. (2012). Soft tissue response to zirconia and titanium implant abutments: An in vivo within-subject comparison. J. Clin. Periodontol..

[6] Barwacz C.A., Brogden K.A., Stanford C.M., Dawson D.V., Recker E.N., Blanchette D. (2015). Comparison of pro-inflammatory cytokines and bone metabolism mediators around titanium and zirconia dental implant abutments following a minimum of 6 months of clinical function. Clin. Oral Implant. Res..

[7] Martins D., Couto R., Fonseca E.M.M., Carreirasa A.R. (2021). Numerical analysis of the mechanical stimuli transferred from a dental implant to the bone. J. Comput. Appl. Res. Mech. Eng..

[8] Brizuela A., Herrero-Climent M., Rios-Carrasco E., Rios-Santos J.V., Pérez R.A., Manero J.M., Gil Mur J., Herrero-Climent M. (2019). Influence of the Elastic Modulus on the Osseointegration of Dental Implants. Materials.

[9] Szmukler-Moncler S., Salama H., Reingewirtz Y., Dubruille J.H. (1998). Timing of loading and effect of micromotion on bone-dental implant interface: Review of experimental literature. J. Biomed. Mater. Res..

[10] Glauser R., Sennerby L., Meredith N. (2004). Resonance frequency analysis of im- plants subjected to immediate or early functional occlusal loading. Successful vs. failing implants. Clin. Oral Implants Res..

[11] Meredith N., Alleyne D., Cawley P. (1996). Quantitative determination of the stability of the implant-tissue interface using resonance frequency analysis. Clin. Oral Implant. Res..

[12] Albrektsson T. (1998). Osseointegration: Historic background and current concepts. J. Clin. Periodontol. Implant Dent..

[13] Brizuela-Velasco A., Álvarez-Arenal Á., Gil-Mur F.J., Herrero-Climent M., Chávarri-Prado D., Chento-Valiente Y., Dieguez-Pereira M. (2015). Relationship Between Insertion Torque and Resonance Frequency Measurements, Performed by Resonance Frequency Analysis, in Micromobility of Dental Implants: An In Vitro Study. Implant. Dent..

[14] Trisi P., Carlesi T., Colagiovanni M. (2010). Implant stability quotient (ISQ) vs. direct in vitro measurement of primary sta- bility (micromotion): Effect of bone density and insertion torque. J. Osteol. Biomat..

[15] Pagliani L., Sennerby L., Petersson A., Verrocchi D., Volpe S., Andersson P. (2013). The relationship between resonance frequency analysis (RFA) and lateral displacement of dental implants: An in vitro study. J. Oral Rehabil..

[16] Barewal R.M., Stanford C., Weesner T.C. (2012). A randomized controlled clinical trial comparing the effects of three loading protocols on dental implant stability. Int. J. Oral Maxillofac. Implant..

[17] Ostman P.-O., Hellman M., Sennerby L. (2005). Direct implant loading in the edentulous maxilla using a bone density-adapted surgical protocol and primary implant stability criteria for inclusion. Clin. Implant. Dent. Relat. Res..

[18] Esposito M., Grusovin M.G., Maghaireh H., Worthington H.V. (2013). Interventions for replacing missing teeth: Different times for loading dentalimplants. Cochrane Database Syst. Rev..

[19] Hanawa T. (2020). Zirconia versus titanium in dentistry: A review. Dent. Mater. J..

[20] Lekholm U., Zarb G.A., Branemark P.I., Zarb G.A., Albrektsson T. (1985). Patient selection and preparation. Tissue Integrated Prsotheses: Osseointegration in Clinical Dentistry.

[21] Tricio J., van Steenberghe D., Rosenberg D., Duchateau L. (1995). Implant stability related to insertion torque force and bone density: An in vitro study. J. Prosthet. Dent..

[22] Tabassum A., Meijer G.J., Wolke J.G.C., Jansen J.A. (2010). Influence of surgical technique and surface roughness on the primary stability of an implant in artificial bone with different cortical thickness: A laboratory study. Clin. Oral Implant. Res..

[23] Dos Santos M.V., Elias C.N., Lima J.H.C. (2009). The Effects of Superficial Roughness and Design on the Primary Stability of Dental Implants. Clin. Implant. Dent. Relat. Res..

[24] Watanabe M., Hattori Y., Satoh C. (2005). Biological and biomechanical perspectives of normal dental occlusion. Int. Congr. Ser..

[25] Elias C.N., Rocha F.A., Nascimento A.L., Coelho P.G. (2012). Influence of implant shape, surface morphology, surgical technique and bone quality on the primary stability of dental implants. J. Mech. Behav. Biomed. Mater..

[26] Ibrahim A., Heitzer M., Bock A., Peters F., Möhlhenrich S.C., Hölzle F., Modabber A., Kniha K. (2020). Relationship between Implant Geometry and Primary Stability in Different Bony Defects and Variant Bone Densities: An In Vitro Study. Materials.

[27] Karl M., Scherg S., Grobecker-Karl T. (2017). Fracture of Reduced-Diameter Zirconia Dental Implants Following Repeated Insertion. Int. J. Oral Maxillofac. Implant..

[28] Delgado-Ruiz R.A., Marković A., Calvo-Guirado J.L., Lazić Z., Piattelli A., Boticelli D., Maté-Sánchez J.E., Negri B., Ramírez-Fernández M.P., Mišić T. (2014). Implant stability and marginal bone level of microgrooved zirconia dental implants: A 3-month experimental study on dogs. Vojnosanit. Pregl..

[29] Dubruille J.H., Viguier E., Le Naour G., Dubruille M.T., Auriol M., Le Charpentier Y. (1999). Evaluation of combinations of titanium, zirconia, and alumina implants with 2 bone fillers in the dog. Int. J. Oral Maxillofac. Implant..

[30] Aboushelib M.N., Salem N.A., Taleb A.L.A., El Moniem N.M.A. (2013). Influence of Surface Nano-Roughness on Osseointegration of Zirconia Implants in Rabbit Femur Heads Using Selective Infiltration Etching Technique. J. Oral Implant..

[31] Park Y.-S., Chung S.-H., Shon W.-J. (2013). Peri-implant bone formation and surface characteristics of rough surface zirconia implants manufactured by powder injection molding technique in rabbit tibiae. Clin. Oral Implant. Res..

[32] Salem N.A., Taleb A.L.A., Aboushelib M.N. (2012). Biomechanical and Histomorphometric Evaluation of Osseointegration of Fusion-Sputtered Zirconia Implants. J. Prosthodont..

[33] Schierano G., Mussano F., Faga M.G., Menicucci G., Manzella C., Sabione C., Genova T., Von Degerfeld M.M., Peirone B., Cassenti A. (2015). An Alumina Toughened Zirconia Composite for Dental Implant Application: In Vivo Animal Results. BioMed Res. Int..

[34] Siddiqi A., Duncan W.J., De Silva R.K., Zafar S. (2016). One-Piece Zirconia Ceramic versus Titanium Implants in the Jaw and Femur of a Sheep Model: A Pilot Study. BioMed Res. Int..

[35] Stadlinger B., Hennig M., Eckelt U., Kuhlisch E., Mai R. (2010). Comparison of zirconia and titanium implants after a short healing period: A pilot study in mini-pigs. Int. J. Oral Maxillofac. Surg..

[36] Lee B.-C., Yeo I.-S., Kim D.-J., Lee J.-B., Kim S.-H., Han J.-S. (2013). Bone formation around zirconia implants combined with rhBMP-2 gel in the canine mandible. Clin. Oral Implant. Res..

[37] Koch F.P., Weng D., Kramer S., Biesterfeld S., Jahn-Eimermacher A., Wagner W. (2010). Osseointegration of one-piece zirconia implants compared with a titanium implant of identical design: A histomorphometric study in the dog. Clin. Oral Implant. Res..

[38] Payer M., Heschl A., Koller M., Arnetzl G., Lorenzoni M., Jakse N. (2014). All-ceramic restoration of zirconia two-piece implants—A randomized controlled clinical trial. Clin. Oral Implant. Res..

[39] Osman R.B., Swain M.V., Atieh M., Ma S., Duncan W. (2013). Ceramic implants (Y-TZP): Are they a viable alternative to titanium implants for the support of overdentures? A randomized clinical trial. Clin. Oral Implant. Res..

[40] Grassi F.R., Capogreco M., Consonni D., Bilardi G., Buti J., Kalemaj Z. (2015). Immediate occlusal loading of one-piece zirconia implants: Five-year radiographic and clinical evaluation. Int. J. Oral Maxillofac. Implant..

